# Position of the Body

**Published:** 1881-08

**Authors:** 


HALL'S
### JOURNAL OF HEALTH
AND
MISCELLANY.

E. H. GIBBS, A. M., M. D. - - EDITOR.

Founded in 1854, and published Monthly without Interruption since that Time.

### POSITION OF THE BODY.

WE all admire the erect form and graceful movements of a person in perfect health; because these are as nature intended. A short man who carries himself erect seems much taller than he is.

Louis XIV.—called the Grand Monarch—was a man of commanding presence, and popularly believed to be almost a giant in stature. When he died his attendants, and even his intimate friends, were surprised to find, instead of the tall form, a body below the medium height. There is more difference in the stature of individuals whom we meet in the street than there seems to be; for those who are short hold up their heads so that they may seem as tall as possible, while the overgrown seek to conceal their altitude by depressing their necks and shoulders. Nature intended that we should carry the points of the shoulders on a line with the neck, or even a little back of it, so that the chest may come out round and full, thus giving heart and lungs ample room in which to do their work. Frequent or long-continued deviations from this natural position result injuriously. The habit of sleeping with the hands above the head cannot be too severely condemned. The temperature of the sleeping-room is almost always lower toward morning, thus exposing the person to cold; but two far more serious results follow this habit: one is the obstruction of the circulation on account of the unnatural position of the arms, and the pressure of the stomach and bowels upon the large artery, which is located along the inner side of the backbone; the other is the direct pressure which the contraction of the chest exerts on the heart and lungs. No person can sleep in this position half an hour without a sense of painful fatigue on waking, which may last for days, and always does positive, and often permanent injury. Those who have the care of children should be watchful lest they fall into the habit of sleeping in that position. There are thousands of men and women to-day suffering from this habit who do not suspect the cause of their ill-health.

Many persons are in the habit of "reading themselves to sleep." Aside from the danger of setting the bed on fire, which should be sufficient to deter a sensible person from a practice so hazardous to the lives of the household, the injury to the eyes is serious and often irreparable, since the impingment of the blood toward the head, with the strain upon the eyes—unavoidable while reading in that position—results in congestion, causing a person to look as if he had been weeping.

There is another position of the body, formed by sitting with the feet elevated above the head, which is not only a gross violation of good manners, but may cause injury to the kidneys and spine. The spinal cord is an extension of the brain, and united with it at its base, and passes through the spinal column to the lower portion of the back. It is therefore plain to be seen that the position above referred to must exert a strain on the spinal cord as well as undue pressure on the kidneys.

In those lounging places, called "reading rooms," connected with hotels, we often see men with their feet elevated as high as their heads. As a people, we doubtless deserve much of the reproach for ill breeding that foreigners cast upon us. No foreigner of refinement would be guilty of such a breach of good manners. The Greek word for man is *anthropos*, meaning uplifted countenance, in contra-distinction to the lower order of animals whose gaze is downward toward the earth. Man's physical nature does not greatly differ from that of other animals, but there the resemblance ceases. The body is not the man. It is a dwelling leased to him for a season by the Great Architect of the universe. It is a temple of marvelous construction intrusted to his keeping, with the command not to abuse it under pains and penalties. Every abuse, every neglect, every rash act is a breach of this sacred trust that will be surely punished. The Greeks and Romans made cleanliness and attention to health part of their religion, and their example is worthy of general adoption.

The great affairs of the world and most of its wealth are to-day mainly in the hands of men who regard "health as a duty" and who live up to their belief.

---

It is only in Anglo-Saxon countries that hotels supply their guests with soap.

